# A Case of IDH‐Mutant Astrocytoma Harboring an IDH2 R172_H173delinsSN Variant

**DOI:** 10.1111/neup.70048

**Published:** 2026-02-24

**Authors:** Thomas Auen, Jie Chen, Nicole Shonka, Sahara Cathcart

**Affiliations:** ^1^ Department of Pathology, Microbiology, and Immunology University of Nebraska Medical Center Omaha Nebraska USA; ^2^ Department of Internal Medicine, Division of Oncology and Hematology University of Nebraska Medical Center Omaha Nebraska USA

**Keywords:** IDH (Isocitrate dehydrogenase)‐mutant astrocytoma, IDH2 (Isocitrate dehydrogenase 2), IDH‐mutant glioma, non‐canonical

## Abstract

IDH‐mutant gliomas most commonly harbor the canonical *IDH1* p.R132H mutation, followed by less common mutations involving *IDH1* p.R132 or *IDH2* p.R172 codons. We present a case of a 32‐year‐old male found to have a left temporal brain tumor with regional enhancement on brain MRI, for which he underwent resection. Histologic sections showed an infiltrating astrocytic tumor with increased mitotic activity and elevated Ki67 (MIB1) labeling. The tumor was negative for IDH1 p.R132H mutant protein expression with retained ATRX expression. 1p/19q was intact by FISH analysis, and next‐generation sequencing identified a previously unreported *IDH2* (p.R172_H173delinsSN) likely pathogenic variant. A diagnosis of astrocytoma, IDH‐mutant, CNS WHO grade 3 was rendered. Subsequent tumor methylation profiling performed at the National Institutes of Health matched with high confidence to the class “IDH glioma, subclass astrocytoma” and confirmed lack of *MGMT* promoter hypermethylation. He received adjuvant concurrent radiation and temozolomide. Surveillance brain MRI at 32 months post resection demonstrated no evidence of interval tumor progression. We present a case of IDH‐mutant astrocytoma with a somewhat atypical molecular presentation, including a previously uncharacterized *IDH2* mutation, retained ATRX expression, and lack of *MGMT* promoter hypermethylation. Though it has not been biochemically or functionally validated, tumor methylation profiling is supportive of this previously uncharacterized *IDH2* variant as tumorigenic.

**Abbreviations**
ATRX: Alpha‐thalassemia/mental retardation, X‐linked
CDKN2A: Cyclin‐dependent kinase inhibitor 2A
CNS: Central nervous system
EGFR: Epidermal growth factor receptor
FISH: Florescence in situ hybridization
FLAIR: Fluid‐attenuated inversion recovery
H3F3A: H3 histone family member 3A
IDH: Isocitrate dehydrogenase
IDH1: Isocitrate dehydrogenase 1
IDH2: Isocitrate dehydrogenase 2
MGMT: O6‐methylguanine‐DNA methyltransferase
MRI: Magnetic resonance imaging
MYCL: MYCL proto‐oncogene, bHLH transcription factor
NADPH: Nicotinamide adenine dinucleotide phosphate, reduced form
NCBI: National Center for Biotechnology Information
NCI: National Cancer Institute
NGS: Next‐generation sequencing
PTEN: Phosphatase and tensin homolog deleted on chromosome 10
TERT: Telomerase reverse transcriptase
TP53: Tumor protein p53
UMAP: Uniform manifold approximation and projection
WHO: World Health Organization

## 1 Introduction

IDH‐mutant astrocytoma is an *IDH1*‐ or *IDH2*‐mutant adult‐type infiltrating glioma with the absence of 1p/19q codeletion, frequent *ATRX* (alpha‐thalassemia/mental retardation, X‐linked) and *TP53* (tumor protein p53) mutations, and frequent *MGMT* (O6‐methylguanine‐DNA methyltransferase) promoter hypermethylation. The vast majority, reportedly 89%–93%, of IDH‐mutant gliomas harbor the canonical *IDH1* p.R132H mutation, followed by *IDH1* p.R132C, p.R132G, p.R132S, p.R132L, and p.R132K. *IDH2* mutations are relatively uncommon, accounting for approximately 3% of all IDH1 and IDH2 mutations in glioma, occurring more frequently in oligodendrogliomas, with p.R172K being the most frequent, and rare cases harboring p.R172L, p.R172M, p.R172W, p.R172S, p.R172G, and p.R172I [1, 2], and further exceptionally rare reports of gliomas harboring *IDH2* p.P158L and p.P162S [3]. We present a case of astrocytoma, IDH‐mutant, CNS WHO grade 3 with a previously unreported *IDH2* (p.R172_H173delinsSN) variant confirmed by sequencing studies and supported by DNA methylation profiling.

## 2 Case Presentation

**FIGURE 1:**
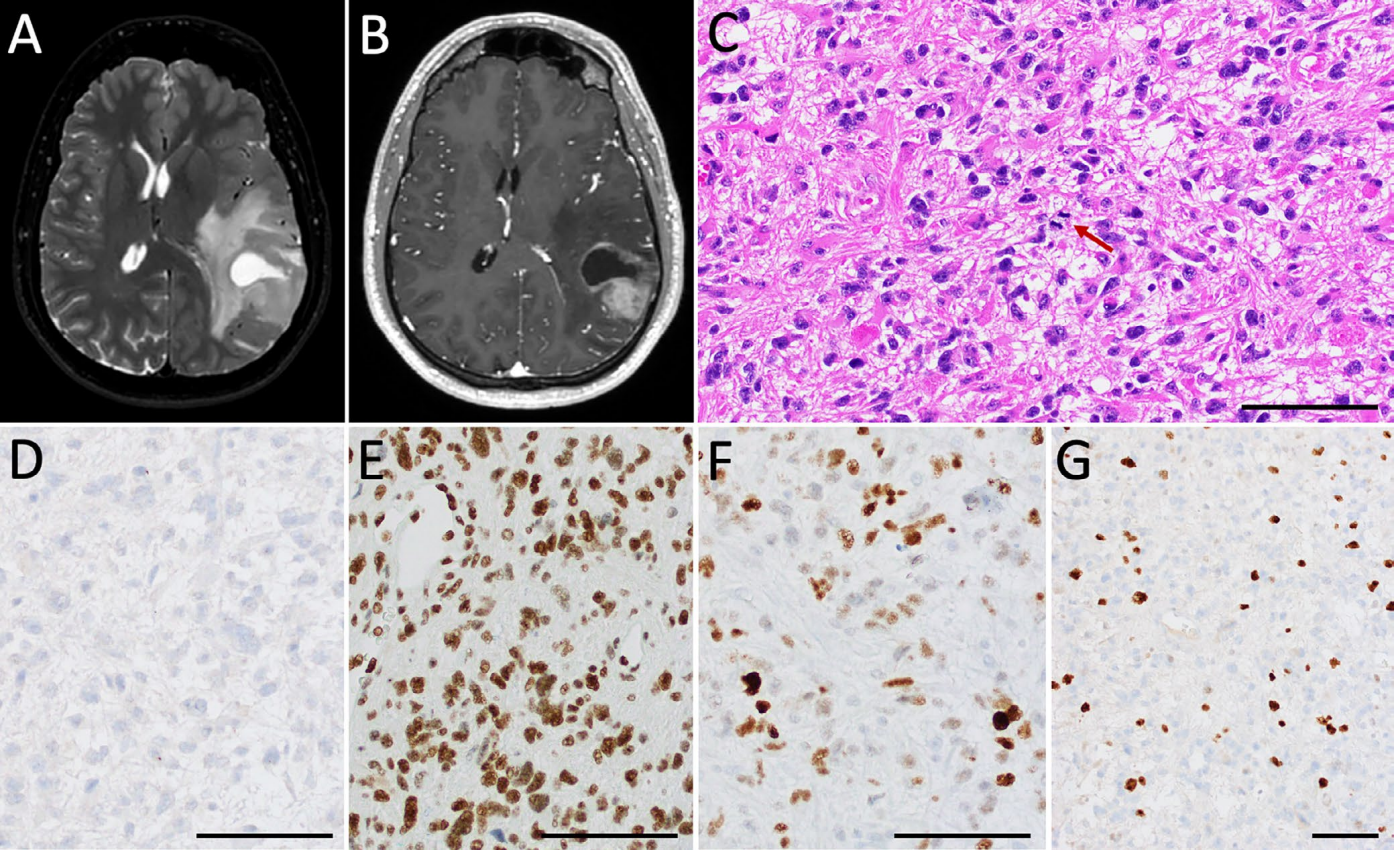
Preoperative brain MRI demonstrated a T2/FLAIR‐hyperintense solid and cystic, mass (A, T2) in the left posterior temporoparietal lobe with nodular contrast enhancement (B, T1 postcontrast). Microscopic examination demonstrated an infiltrating astrocytoma with hyperchromasia, irregular nuclear borders, and increased mitotic activity (C, H&E, 200×, mitosis highlighted by red arrow) without definitive vascular proliferation or necrosis identified. Tumor cells were negative for IDH1 p.R132H mutant protein expression (D) with retained nuclear ATRX expression (E) and overexpression of p53 (F). The Ki67 (MIB1) proliferative index was elevated at approximately 10%–15% (G). Black bars (C–G) = 50 μm.

**FIGURE 2:**
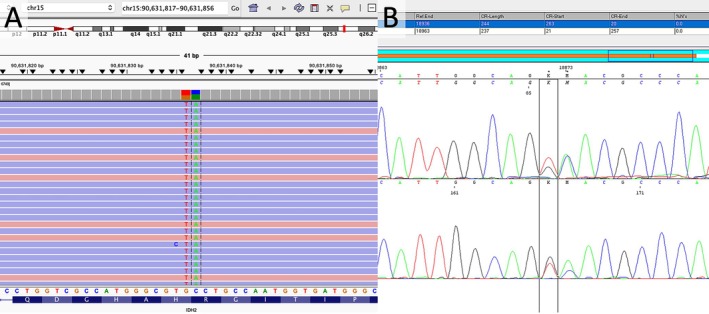
Next‐generation sequencing (Precision Oncology Profile 300, adapted from Illumina TruSight Oncology 500 panel, https://www.testmenu.com/nebraska/Tests/1224493) identified a novel *IDH2* (c.516_517delGCinsTA; p.R172_H173delinsSN) variant at a VAF of 56% (A), which was confirmed by dideoxy sequencing (B).

The patient is a 32‐year‐old male who presented with headaches, nausea, and vomiting and was found to have a 4.2‐cm, T2/FLAIR hyperintense, solid and cystic left posterior temporal mass with regional enhancement on magnetic resonance imaging (MRI) (Figure 1A,B). He underwent left frontotemporal parietal craniotomy for resection with awake cortical language mapping. Neuropathological examination demonstrated an infiltrating astrocytoma with nuclear irregularity and hyperchromasia (Figure 1C–G). A prominent delicate vascular network was present without definitive microvascular proliferation. No necrosis was identified. Foci of perivascular inflammation were present. At least 8 mitoses per 10 high power fields (3.5 per mm^2^) were identified, and the Ki67 proliferation index was elevated at approximately 10%–15%. By immunohistochemistry, the tumor was negative for IDH1 p.R132H mutant protein expression with retained ATRX expression. Approximately 70% of tumor cells had weak to moderate nuclear p53 overexpression. Fluorescence in situ hybridization (FISH) analysis demonstrated intact chromosome 1p36 and 19q13 loci and absence of *CDKN2A* (cyclin‐dependent kinase inhibitor 2A) (9p21) homozygous deletion. Gain of the 9p21 locus was seen in most tumor cells. Next‐generation sequencing (NGS; Precision Oncology Profile 300, adapted from Illumina TruSight Oncology 500 panel, https://www.testmenu.com/nebraska/Tests/1224493) was performed with an estimated tumor purity of approximately 80%. Likely pathogenic mutations were identified in *TP53* (p.H179R and c.994‐2A > G) at variant allele frequencies (VAF) of 45% and 46%, respectively, as well as a previously uncharacterized *IDH2* (c.516_517delGCinsTA; p.R172_H173delinsSN) variant at a VAF of 56% (Figure 2A). *MYCL* copy number gain was also identified. Notably, no variants were identified in the *EGFR*, *PTEN*, *TERT, H3F3A*, or *ATRX* genes, nor were other variants in *IDH1* or *IDH2* identified. *MGMT* testing was negative for promoter hypermethylation by pyrosequencing. The identified *IDH2* variant was confirmed by dideoxy (Sanger) sequencing (Figure 2B). To further validate the novel *IDH2* variant, tissue was sent to the NCI/NIH for DNA tumor methylation profiling. The tumor demonstrated a consensus match to the class “IDH glioma, subclass astrocytoma” on the NCI/Bethesda classifier and versions 11b6 and 12b6 of the Heidelberg classifier with high‐confidence scores. Dimensionality reduction with UMAP (Uniform Manifold Approximation and Projection) also placed the tumor in the same class. Additionally, absence of *MGMT* promoter hypermethylation was corroborated. The patient received adjuvant concurrent radiation and temozolomide. Imaging 4 months post resection was concerning for mild progression. He received six additional cycles of temozolomide over the following 6 months, after which MRI demonstrated partial response. MRI at approximately 32 months post‐resection demonstrated no evidence of interval tumor progression.

## 3 Discussion

We present an IDH‐mutant astrocytoma with a somewhat atypical molecular phenotype including a previously uncharacterized *IDH2* (c.516_517delGCinsTA, p.R172_H173delinsSN; NM_002168.2) variant involving a compound substitution of serine (S) and asparagine (N) in place of arginine (R) and histidine (H) at codons 172 and 173, respectively, notably involving the hotspot R172 codon. Additionally, the tumor exhibited retained ATRX expression and lack of *MGMT* promoter methylation, findings each documented in a minority of IDH‐mutant astrocytoma. A literature search of English‐language articles published on PubMed dating through May 2025 (keywords: “IDH” OR “IDH1” OR “IDH2” OR “isocitrate dehydrogenase” AND “glioma” AND “non‐canonical”) yielded no mention of an R172 compound substitution variant among identified non‐canonical *IDH2* mutations in astrocytoma. This novel *IDH2* variant has also not been reported in the ClinVar NCBI database. This described *IDH2* variant has not been functionally validated, and thus its pathogenicity is not unequivocally determined. Though not definitive evidence, tumor methylation profiling with high confidence match to the class “IDH glioma, subclass astrocytoma” offers supportive data that this novel *IDH2* variant is likely tumorigenic and may result in similar biochemical and epigenetic changes as seen in typical IDH‐mutant gliomas. Notably, the *IDH2* R172 wildtype codon is highly conserved across vertebrate species and eukaryotic *IDH2* homologs [4, 5], such that any alterations at this codon would be expected to have significance. In addition, this variant has not been identified in gnomAD browser (gnomad.broadinstitute.org), indicating no known population frequency.

While identification of an *IDH1* or *IDH2* mutation in the setting of glioma is critical for diagnostic accuracy, it has more recently been assessed as a therapeutic marker in gliomas. The IDH1 (cytosolic) and IDH2 (mitochondrial) isoforms of isocitrate dehydrogenase (IDH) convert isocitrate to α‐ketoglutarate to produce NADPH. Mutant forms of IDH further reduce α‐ketoglutarate to the oncometabolite D‐2‐hydroxyglutarate (D‐2‐HG), which results in downstream global DNA hypermethylation patterns, impaired immunity, and gliomagenesis [6]. Vorasidenib, a brain‐penetrant next‐generation dual inhibitor of mutant IDH1 and IDH2, validated using *IDH1* p.R132H and *IDH2* p.R140Q models, was demonstrated to bind analogous allosteric pockets of each mutant protein, respectively, with good potency against both isoforms [7]. It has recently been studied in an international phase 3 trial in grade 2 *IDH1*‐ and *IDH2*‐mutant gliomas (INDIGO trial, NCT04164901) and demonstrated improved median progression‐free survival (27.7 versus 11.1 months) and delayed time to subsequent intervention. Importantly, the vast majority of enrolled test patients harbored the canonical *IDH1* p.R132H mutation, with only small numbers of patients harboring mutations in *IDH1* p.R132C, p.R132G, p.R132L, and p.R132S, as well as only a few patients with *IDH2* p.R172K and p.R172G [8]. With potential expanded future use of therapeutic IDH inhibitors to CNS tumors, recognition and future study of rare *IDH1*/*2* mutations will be clinically important for these select patients to further establish efficacy. Though not a substitution for formal functional validation, this case also highlights how the use of DNA methylation profiling may help classify uncharacterized variants within tumor class‐defining genes.

## Funding

The authors have nothing to report.

## Disclosure

The authors have nothing to report.

## Consent

The patient has given consent for the publication of this case.

## Conflicts of Interest

The authors declare no conflicts of interest.

## Data Availability

The data that support the findings of this study are available on request from the corresponding author. The data are not publicly available due to privacy or ethical restrictions.
